# COVID-19 mRNA Vaccination-Induced Myopericarditis in an Otherwise Healthy Young Male: An Evidence-Based Approach to Differentiating From Perimyocarditis

**DOI:** 10.7759/cureus.59999

**Published:** 2024-05-09

**Authors:** Lekhini Latchupatula, Myles Benayon, Laurie Yang, Javier Ganame, Vikas Tandon

**Affiliations:** 1 Internal Medicine, McMaster University, Hamilton, CAN; 2 Medical School, McMaster University, Hamilton, CAN; 3 Medicine, McMaster University, Hamilton, CAN

**Keywords:** endomyocardial biopsy, cardiac magnetic resonance imaging, pericarditis, myocarditis, perimyocarditis, vaccine, covid-19, myopericarditis

## Abstract

A 29-year-old male, otherwise healthy with no past medical history, presented to the hospital after a two-day history of pleuritic chest pain with a fever. He had received his first dose of the mRNA-1273 coronavirus disease (COVID-19) vaccine (Moderna) two months prior without any adverse reactions. He received his second dose approximately 24 hours before symptom onset and hospital presentation. Work-up was unremarkable for respiratory, autoimmune, and rheumatological etiologies. The patient was found to have electrocardiogram features and symptoms in keeping with pericarditis, C-reactive protein elevation, and a peak high-sensitivity troponin level of 9,992 ng/L suggestive of a component of myocarditis. A dilemma arose regarding whether this patient should be diagnosed with perimyocarditis or myopericarditis, terms often used interchangeably without proper reference to the primary pathology, which can ultimately affect management. A subsequent echocardiogram was unremarkable, with a normal left ventricular systolic function, but cardiac resonance imaging revealed myocardial edema suggestive of myocarditis. Without convincing evidence for an alternative explanation after an extensive work-up of ischemic, autoimmune, rheumatological, and infectious etiologies, this patient was diagnosed with COVID-19 mRNA vaccine-induced myopericarditis. The patient fully recovered after receiving a treatment course of ibuprofen and colchicine. This case explores how the diagnosis of COVID-19 vaccine-induced myopericarditis was made and treated using an evidence-based approach, highlighting its differentiation from perimyocarditis.

## Introduction

Myocarditis and pericarditis, inflammation of the myocardium and pericardium, respectively, are heterogeneous diseases with a wide variety of clinical presentations, severities, and outcomes [1,2]. 

Myopericarditis reflects a primary pericardial involvement, with myocardial involvement as a smaller factor. The European Society of Cardiology (ESC), in 2015, defined the diagnosis of myopericarditis as meeting definite criteria for acute pericarditis but also having elevated biomarkers suggestive of myocardial injury [2]. Furthermore, myopericarditis patients must show no new-onset impairment of left ventricular (LV) function and normal wall motion in cardiovascular magnetic resonance imaging (CMR) or echocardiography [2]. Alternatively, if new LV dysfunction, either focal or diffuse, is present, this is more suggestive of myocarditis being the primary entity and would be defined as perimyocarditis [2]. 

While the terms “myopericarditis” and “perimyocarditis” have historically been used interchangeably, recent studies sought to clarify this terminology [3]. This has clinical implications in treatment since myopericarditis is treated similarly to pericarditis with non-steroidal anti-inflammatory drugs (NSAIDs) and colchicine, whereas in perimyocarditis, guideline-directed medical therapy may be initiated given the presence of LV dysfunction. Further, there is evidence in past animal model studies suggesting that NSAIDs could worsen inflammation and mortality in myocarditis. The applicability of these findings in humans and the value of reduced doses of NSAIDs in the treatment of myocarditis or perimyocarditis is highly debated. There is also not sufficient evidence to definitively recommend colchicine in perimyocarditis, but evidence for colchicine is much more substantial in myopericarditis [2].

Viral infections are the most frequently presumed cause of myocarditis and pericarditis in resource-abundant North American and European countries. Commonly implicated pathogens include adenovirus, enterovirus, human herpesvirus six, and parvovirus. Both disease processes can also be secondary to autoimmune disorders or be induced by vaccination for a wide variety of viruses [1-4]. Since the coronavirus disease 2019 (COVID-19) pandemic, COVID-19 vaccination has been identified as an increasingly reported cause of myopericarditis, especially in young males [5]. 

In this report, we present a case of an otherwise healthy young male who presented with significant features of both pericarditis and myocarditis after COVID-19 vaccination. We explore the diagnostic assessments and criteria for differentiating myopericarditis from perimyocarditis and inform subsequent management.

This article was previously presented as an abstract at the 2021 Rocky Mountain Internal Medicine Conference on November 19, 2021.

## Case presentation

A 29-year-old male presented to the hospital emergency room (ER) with a two-day history of retrosternal pleuritic chest pain. He had no prior medical history. He was not on any medications at home and had no known drug allergies.

His first dose of the mRNA-1273 (Moderna) COVID-19 vaccine was administered two months prior without any adverse reactions. The patient was feeling well on the day he received his second dose of the same vaccine. He had no recent infections of any kind. Approximately 24 hours after that, he developed general fatigue, pleuritic retrosternal chest pain that did not radiate, and shortness of breath on exertion. He denied any history of palpitations, orthopnea, paroxysmal nocturnal dyspnea, leg swelling, rhinorrhea, odynophagia, or cough. He was a lifelong non-smoker and had no family history of cardiovascular or other diseases.

**Figure 1:**
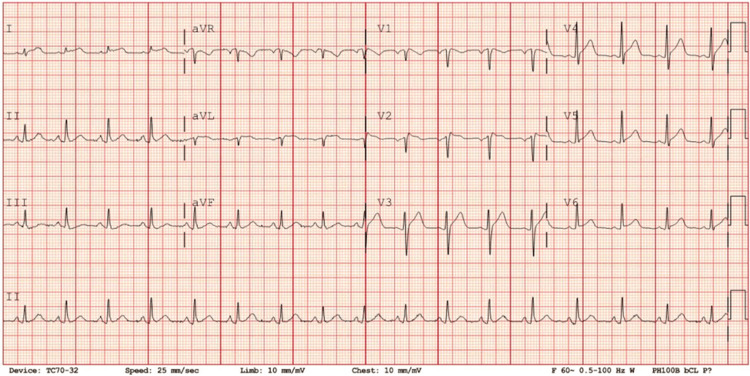
The patient’s initial electrocardiogram in the emergency department revealed sinus tachycardia with a heart rate of 101 beats per minute, PR depression, and diffuse ST-elevation suggestive of pericarditis.

Upon arrival at the ER, the patient was febrile at 38.2 degrees Celsius, but his vitals were otherwise normal. His cardiovascular exam was unremarkable, with no pericardial rub and his chest was clear to auscultation. There was no peripheral edema or increased jugular venous pressure. An electrocardiogram (ECG) revealed sinus tachycardia with a heart rate of 101, PR depression, and diffuse ST-elevation consistent with pericarditis (Figure 1). 

Laboratory investigations (Table 1) were significant for an elevated high-sensitivity troponin that peaked at 9,992 ng/L (reference value: ≤ 30 ng/L), a mildly elevated N-terminal pro-B-type natriuretic peptide at 163 ng/L (reference value: ≤ 126 ng/L), and an elevated C-reactive protein at 37 mg/L (reference < 10 mg/L). An extensive laboratory work-up for autoimmune, rheumatological, and infectious causes was unremarkable in determining the etiology. His chest x-ray was normal and showed no cardiomegaly, consolidations, or signs of pulmonary edema. 

**Table 1:** The patient’s pertinent blood work during his hospital admission. COVID-19: coronavirus disease 2019

Test	Result	Reference Range
Autoimmune & Inflammatory		
Antinuclear Antibody	Negative	Not Applicable
C3 Complement (g/L)	1.70	0.79-1.52
C4 Complement (g/L)	0.80	0.16-0.38
Cyclic Citrullinated Peptide Antibody (Units)	<18	<20
C-Reactive Protein (mg/L)	37	<10
Rheumatoid Factor (IU/mL)	<20	<20
Cardiac Markers		
Creatine Kinase (U/L)	431	45-250
N-Terminal Pro B-Type Natriuretic Peptide (ng/L)	163	<126 ng/L
Troponin l-High-Sensitivity (ng/L) - Peak	9,992	<35
Coagulation Studies		
D-Dimer (μg/L)	<270	<500
International Normalized Ratio	1.2	0.8-1.2
Complete Blood Count		
Hemoglobin (g/L)	134	130-180
Leukocytes (x10^9/L)	9.7	4.0-11.0
Platelets (x10^9/L)	313	150-400
Electrolytes		
Bicarbonate (mmol/L)	28	22-30
Chloride (mmol/L)	99	95-110
Magnesium (mmol/L)	0.96	0.70-1.10
Phosphate (mmol/L)	1.16	0.90-1.52
Potassium (mmol/L)	4.1	3.5-5.2
Sodium (mmol/L)	140	135-145
Liver Enzymes		
Alanine Transaminase (U/L)	34	0-49
Alkaline Phosphatase (U/L)	75	38-126
Aspartate Aminotransferase (U/L)	84	18-54
Gamma-glutamyl Transferase (U/L)	24	<65
Microbiology		
COVID-19 RNA	Negative	
Cytomegalovirus Immunoglobulin G Antibody	Non-reactive	
Epstein-Barr Virus Nuclear Antigen Immunoglobulin G	Non-reactive	
Hepatitis B Surface Antigen	Non-reactive	
Human Immunodeficiency Virus	Non-reactive	
Parvovirus B19 Serology	Non-reactive	
Query Fever Serology	Non-reactive	
Other Serum Chemistries		
Albumin (g/L)	48	42-50
Erythrocyte Sedimentation Rate (mm/h)	12	0-15
Ferritin (μg/L)	148	>30
Lactate Dehydrogenase (U/L)	218	120-250
Random Glucose (mmol/L)	6.1	4.0-7.8
Thyroid Stimulating Hormone (mlU/L)	4.58	0.47-4.68
Renal Function		
Creatinine (umol/L)	84	60-110
Estimated Glomerular Filtration Rate (ml/min/1.73m2)	108	>60
Urea (mmol/L)	4.3	3.5-8.6

There was initial debate among team members about whether NSAIDs should be started given his markedly elevated troponin and concern if there was a primary myocarditis component as opposed to pericarditis. A subsequent transthoracic echocardiogram was unremarkable, revealing an estimated LV ejection fraction (LVEF) of 67% with no regional wall motion abnormalities (RWMAs), diastolic dysfunction, or pericardial effusion. Though the patient’s presentation was consistent with a primary component of pericarditis, he had a higher-than-expected peak troponin value, suggesting an element of myocardial involvement. His echocardiogram demonstrated no clear features of myocarditis being the primary entity, such as LV dysfunction or RWMAs; therefore, a diagnosis of myopericarditis was made. 

An angiogram was considered but not pursued as the patient had a low pretest probability for coronary disease or acute coronary syndrome given the absence of coronary disease risk factors and his young age. An endomyocardial biopsy (EMB) was also considered, but given his clinical stability, a non-invasive modality for further investigation was preferred, and a CMR was ordered.

He was started on a three-month course of oral colchicine 0.6 mg twice daily and a tapering course of oral ibuprofen for treatment. His taper consisted of a three-week course of ibuprofen: 400 mg three times daily for the first week, twice daily for the second week, and once daily for the third week.

**Figure 2:**
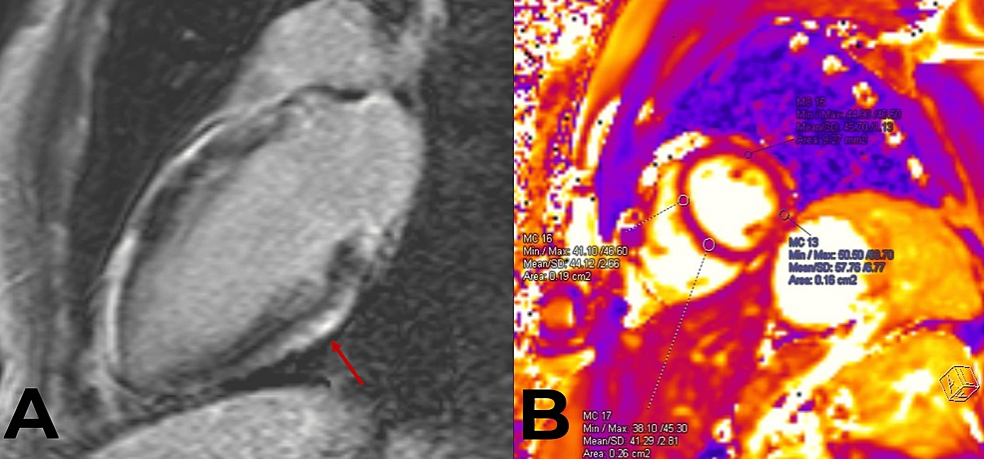
Panel A shows predominantly epicardial delayed enhancement (arrow) of the basal and mid-inferior wall. Panel B shows the image used to calculate T2 mapping values.

The patient’s symptoms resolved within 24 hours of presentation to the hospital, and the patient was asymptomatic and stable at the time of discharge after a two-day admission. A referral to outpatient rheumatology was made at discharge to follow up on some of his pending rheumatological investigations, but these were ultimately unremarkable. He underwent outpatient CMR approximately one week later, which revealed a left and right ventricular ejection fraction of 56% and 44%, respectively. The T2 mapping values were in the upper range of normal in the basal inferolateral segment and minimally elevated in the mid inferolateral lateral segment (Figure 2). There was also epicardial late gadolinium enhancement (LGE) in the basal to mid inferior and inferolateral myocardial segments. Overall, these findings were suggestive of myocardial edema, which is consistent with an element of myocardial involvement.

Outpatient rheumatology consultation revealed no concerns for a rheumatological or autoimmune cause to his presentation. His cardiology and rheumatology practitioners concluded that given the temporal association of his symptom onset with his second COVID-19 vaccination and an absence of other possible etiologies after extensive investigations, his diagnosis would be COVID-19 vaccine-induced myopericarditis. 

Three months following the patient’s hospital discharge, he was seen in follow-up at his outpatient cardiology clinic. He completed his colchicine and ibuprofen courses and continued to be asymptomatic. A repeat echocardiogram was normal. Given his low risk for severe COVID-19 infection and potential for recurrence of myopericarditis, he was advised against future COVID-19 vaccination with the caveat that these recommendations may change based on emerging evidence over time.

## Discussion

This case highlights myopericarditis as a rare complication of COVID-19 vaccination, especially in younger males such as this patient, and the process of distinguishing this from perimyocarditis [5]. Vaccine-induced myopericarditis is not a new phenomenon to the COVID-19 vaccine and has occurred with other vaccinations as well. Through the United States National Passive Vaccine Safety Surveillance System, a 2018 analysis revealed 708 reports between 1990 and 2018 of vaccine-induced myopericarditis. Among these cases, 79% were male, and cases were most commonly reported after smallpox and anthrax vaccination [6]. 

In recent years since the pandemic, several studies have been published documenting myocarditis and pericarditis after COVID-19 vaccination. In a United States retrospective cohort study, a total of 411 events of myocarditis, pericarditis, or both were observed among approximately 15 million people aged 18-64 years of age. These events occurred most frequently after the second COVID-19 vaccine dose and in men aged 18-25 [7]. Similarly, a Canadian study examining mRNA-1273 and BNT162b2 (Pfizer-BioNTech) COVID-19 vaccinations also found the risk of myocarditis or pericarditis to be several times higher in young males aged 18-29 [8]. The incidence of myocarditis has been estimated to be 2.13 per 100,000 among persons within 42 days of COVID-19 vaccination, again most commonly among young males [9]. 

Several case reports have been reported on myopericarditis post-COVID-19 vaccination, most commonly among young males after their second dose [10-13]. A systematic review of 23 observational studies identified that 90.3% of individuals with COVID-19 vaccine-associated myopericarditis are of the male sex. Among these patients, 84% had preserved LV function and 87.2% of patients showed LGE on CMR [10]. Furthermore, patients receiving an mRNA vaccine are approximately four times more likely to develop myopericarditis than those given non-mRNA vaccines [5]. The literature’s identification of being a young male, using an mRNA-based vaccine, and second COVID-19 vaccination dose as high-risk factors are all consistent with our case study’s patient, providing further support for his diagnosis of COVID-19 vaccine-induced myopericarditis.

In this case, several guidelines informed our clinical decision-making and subsequent diagnosis of myopericarditis over perimyocarditis. The patient’s ECG changes and chest pain characterization met the diagnostic criteria for acute pericarditis as outlined by the ESC, which specifies a patient must exhibit a minimum of two out of the four following features: pericarditic chest pain history, pericardial rubs on auscultation, new diffuse ST-elevation or PR depressions on ECG, or new or worsening pericardial effusion [2]. 

Alternatively, the patient’s significantly elevated troponin levels in the context of acute chest pain suggested that the patient may also have myocarditis. The ESC outlined diagnostic criteria for clinically suspected myocarditis in their 2013 report [1]. These guidelines specify that at least one criterion from clinical presentation and one from diagnostic criteria must be met in the absence of angiographically detectable coronary artery disease and pre-existing cardiovascular or extra-cardiac causes. Clinical criteria included acute chest pain, new-onset dyspnea or fatigue, palpitations, aborted sudden cardiac death, syncope, or unexplained cardiogenic shock or arrhythmia symptoms. Diagnostic criteria included a variety of cardiac electrical abnormalities, elevated troponin markers, structural and functional abnormalities on cardiac imaging, and edema or LGE typical of myocarditis on CMR [1]. 

The gold standard test for a definite diagnosis of myocarditis is still EMB to guide diagnosis and management; however, it is not routine practice for all patients with suspected myocarditis to undergo an EMB, and various guidelines debate its use [1,14,15]. In 2016, the American Heart Association (AHA) released a statement illustrating an algorithm to guide EMB use that would balance clinical impact and safety [16]. Specifically, the AHA strongly recommended EMB if a patient presents with acute myocarditis requiring inotropic or mechanical circulatory support, heart block consistent with Mobitz type II or higher grade, symptomatic or sustained ventricular tachycardia, or failed to respond to guideline-directed medical management within one to two weeks [15,16]. If this criterion is not satisfied, CMR is recommended [16]. Therefore, based on this algorithm and our patient’s overall clinical stability, he did not undergo EMB, and instead, a CMR was obtained.

If there is a clinical suspicion of myocarditis, coronary angiography should be considered in all patients [1]. However, for young patients with acute myocardial inflammation but no coronary disease risk factors, an alternative diagnosis is significantly more probable than an ischemic etiology [17]. Given his young age, absence of risk factors, and the high suspicion that his acute presentation was vaccine-induced, no angiogram was pursued in our patient. In these particular cases, CMR can be a first-line investigation for further diagnostic clarity, and ESC guidelines maintain CMR as a Class I recommendation for assessing myocarditis [17]. 

CMR allows for the characterization of the myocardial tissue to support the diagnosis of myocarditis and identify features of myocardial edema without invasive means such as EMB. Novel CMR T2 mapping has a high diagnostic accuracy for acute myocardial edema. Furthermore, the 2018 Lake-Louise Criteria outlines the diagnostic criteria for myocardial inflammation on CMR, which suggests strong evidence of acute myocardial inflammation if both T1 and T2 criteria are met [17]. T1 criterion is considered to be met if there is (i) an increase in native T1 relaxation times, (ii) an increase in extracellular volume, or (iii) a presence of non-ischemic LGE patterns. The T2 criterion is met if there are (i) increased T2 relaxation times or (ii) a presence of regional high T2 signal intensities on T2-weighted images or an increased global T2 signal intensity ratio [17].

While this case’s patient technically did not meet the ESC 2013 myocarditis criteria due to the absence of an angiogram, he did meet the CMR Lake-Louise criteria given his regional LGE and elevated T2 signal intensity [1,17]. In combination with an elevated troponin and acute chest pain, this strongly suggests the patient had a component of myocarditis in addition to meeting the diagnostic criteria for pericarditis. Given that his echocardiogram confirmed the absence of LV dysfunction or RWMAs and the highest evidence-based diagnostic certainty was for pericarditis over myocarditis, this patient was diagnosed with myopericarditis instead of perimyocarditis [2]. Lastly, since the patient responded well to treatment, had high-risk demographics being a young male, and there was an absence of evidence for other etiologies, his diagnosis was most likely COVID-19 mRNA vaccine-induced myopericarditis, particularly in the setting of a clear temporal relation after specifically receiving his second vaccine dose. His clinical course is consistent with the literature that suggests patients presenting early with COVID-19 vaccine-induced acute pericarditis may evolve into myopericarditis [13].

Pericarditis is classically treated with colchicine and NSAIDs, typically acetylsalicylic acid or ibuprofen [2]. In virus-induced myocarditis, the literature has suggested that NSAIDs may cause increased mortality [2,18]. In a study investigating the effects of salicylates and indomethacin usage in murine models with coxsackievirus B3 myocarditis, investigators found increased mortality, higher viral titres, and worsened pathologic cardiac changes [18]. Myocarditis treatment is mainly dependent on the underlying etiology and managing complications such as heart failure and arrhythmias [1]. Other treatment options that have varying levels of evidence can be considered depending on the underlying etiology such as anti-virals, high-dose intravenous immunoglobulin, and immunosuppressive therapies [1]. Given that the primary component of perimyocarditis is myocarditis, NSAIDs are recommended to be used cautiously or at the lowest possible doses to control symptoms. Furthermore, colchicine lacks sufficient evidence regarding its benefit to be recommended routinely in perimyocarditis [19]. However, there is little evidence to suggest that these conclusions apply to patients with myopericarditis, given the myocardial component is not the main pathology [2]. Corticosteroids for myopericarditis are typically only used in refractory cases [20]. There is no randomized control trial evidence to guide myopericarditis treatment. Thus, the patient was treated with a course of NSAIDs and a three-month course of oral colchicine 0.6 mg twice daily per conventional myopericarditis guidelines [20]. 

With or without treatment, the prognosis in vaccine-associated myopericarditis is overall favourable, but treatment with NSAIDs and colchicine is still recommended [19]. While guidelines vary, myopericarditis patients should be counselled to refrain from heavy physical activity and participation in competitive sports until their symptoms and ECG abnormalities have entirely resolved and potentially up to two weeks after vaccination [5,20]. 

Notably, COVID-19 infection is a risk factor for myopericarditis, pericarditis, and myocarditis [5,9,20]. Myopericarditis more frequently occurs after COVID-19 infection rather than COVID-19 mRNA vaccination [5,9,20]. The decision to pursue repeat COVID-19 mRNA vaccination after mRNA COVID-19 vaccine-induced myopericarditis is nuanced and variable depending on an individual’s risks. Healthcare professionals should take into consideration the potential risks associated with both COVID-19 infection and vaccination, along with the possibility of myopericarditis resulting from the infection itself. Recommendations to patients should be made in collaboration with medical experts and consultants who know the risk factors for vaccine-induced myopericarditis and the increasingly emerging evidence around this topic. High-risk patients could be considered for non-mRNA COVID-19 vaccination as a potential alternative [5,20]. 

Future studies are necessary to investigate the mechanisms as to why mRNA vaccine-induced myopericarditis occurs, especially in those who are high-risk. While many hypotheses regarding these mechanisms have been suggested, none have been definitively proven. In young patients who have previously been exposed to COVID-19, RNA may trigger a bystander effect by activating pre-existing autoreactive cells, as RNA itself is a potent immunogen [20]. Others suggest hyperimmune responses, molecular mimicry between self-antigens and COVID-19 antibodies, and abnormal activation of apoptosis may mediate this phenomenon, but several flaws exist with these hypotheses [20]. 

Further investigations are warranted to examine the incidence of myopericarditis after a third mRNA vaccine and to develop more evidence-based recommendations for individuals diagnosed with COVID-19 vaccine-induced myopericarditis.

## Conclusions

This case report underscores myopericarditis as a rare but notable complication of COVID-19 vaccination, highlighting the essential clinical decision-making process to distinguish it from perimyocarditis for appropriate management. The differentiation between these conditions is crucial to understanding the primary pathology and informing treatment strategies, which we illustrate through evidence-based approaches using several clinical and imaging diagnostic criteria. Overall, given the patient’s several risk factors, fulfilment of pericarditis diagnostic criteria, CMR findings suggestive of myocarditis, elevated troponin levels, absence of LV dysfunction or RWMAs, and temporal association with his vaccination, the patient was diagnosed and treated as COVID-19 vaccine-induced myopericarditis with colchicine and NSAIDs. Further research is necessary to understand the exact mechanisms of COVID-19 vaccine-induced myopericarditis and to develop strategies for its prevention, management, and counselling regarding exercise and future vaccination.
